# Massive Gasoline Ingestion in a 64-Year-Old Female: An Explosive Situation

**DOI:** 10.7759/cureus.13466

**Published:** 2021-02-21

**Authors:** Zachary A Koenig, Grant A Robertson, Nicholas I Koenig, Paige R Durkin, Ryan McCarthy

**Affiliations:** 1 School of Medicine, West Virginia University, Morgantown, USA; 2 School of Dentistry, West Virginia University, Morgantown, USA; 3 Internal Medicine, West Virginia University, Martinsburg, USA

**Keywords:** gasoline ingestion, suicidal behavior

## Abstract

Hydrocarbons from gasoline are toxins that can affect a multitude of organ systems based on the route of chemical intoxication exposure, with a majority involving oral ingestion or inhalation. Data is still incomplete concerning the systemic complications of gasoline ingestion due in part to variability in the chemical composition of various gasoline products.

A 64-year-old female presented to her local emergency department following the ingestion of gasoline in a suicide attempt with altered mental status, hypotension, shortness of breath, tachypnea, sinus tachycardia, coarse rhonchi bilaterally, and hyperactive bowel sounds. Treatment upon admission included intravenous ampicillin/sulbactam, intubation, an intravenous fluid bolus, and ketamine to address the developing hypotension. The patient developed multiorgan failure and acute toxic encephalopathy despite medical interventions and hemodialysis. After four days, comfort care measures were initiated, and the patient passed away.

Gasoline toxicity can have a profound effect on multiple organs based on the chemical properties and the route of exposure. These sequelae can be monitored through patient symptoms as well as radiologic imaging. Early supportive therapy and decontamination are vital in decreasing the morbidity and mortality associated with gasoline ingestion.

## Introduction

Given the psychiatric effects of COVID-19, physicians must be able to recognize and treat many types of suicide attempts. Hydrocarbon exposures represent a clinically important cause of poisoning as there are over 28,000 cases reported annually to United States regional poison control centers [1]. The dose and route of exposure determine which organs are predominantly affected, while the chemical properties of the individual hydrocarbon determine the specific toxicity. The different routes of exposure include ingestion, inhalation, dermal exposure, and parenteral injection, with ingestion being the most common type. The majority of exposures are unintentional and are secondary to children’s exploratory behavior [2]. Most exposures only require supportive care because gagging and coughing limit the amount of hydrocarbons ingested. However, 15% of hydrocarbon exposures are intentional, causing significant toxicity to essentially all body systems [1,2].

Gasoline is an example of an aliphatic hydrocarbon that also contains mixtures of other volatile substances such as toluene, benzene, xylene, and naphthalene [3]. Local effects of gasoline ingestion include mucosal and gastrointestinal irritation which can lead to vomiting [4]. Gasoline has a relatively low surface tension, low viscosity, and high volatility which allow rapid transition between the gaseous and liquid forms. This in turn can cause inhalational injury that occurs simultaneously with ingestion. This creates greater hazard risk for aspiration pneumonitis or pneumonia that leads to absorption across the alveolar mucosal barrier and entry into systemic circulation [3,4].

Still yet, it is only possible to generalize about systemic complications from acute toxicity because of the variable composition of the ingested products. Signs of systemic toxicity include hemolysis, soft tissue necrosis, central nervous system depression, heart sensitization to catecholamines, hepatotoxicity, and acute tubular necrosis. Ancillary effects include infection, hypoxemia, and pneumatocele formation [5]. Once suspected, acute hydrocarbon toxicity requires recognition of cardiac, pulmonary, renal, hepatic, or neurologic complications with rapid initiation of appropriate supportive care to provide the best outcome.

## Case presentation

A 64-year-old female with a past medical history of bipolar disease and emphysema presented to her local rural emergency department via emergency medical services after having ingested a large quantity of commercial gasoline. She was non-adherent to her psychiatric medication regimen and claimed that she attempted suicide multiple times in the past through cutting herself as well as benzodiazepine overdose. Although she was awake and alert, she did not provide any details regarding her current state. She was discovered by her family in her bedroom, where she was covered with bloody cloths and in respiratory distress. Her family reported that they believe the ingestion took place 12 hours prior to her arrival, but they did not know the type of gasoline that was ingested.

Initial examination revealed a respiratory rate of 43, oxygen saturation of 88%, and sinus rhythm with tachycardia. She had coarse rhonchi bilaterally and hyperactive bowel sounds. An odor of gasoline was detected to be coming from the patient.

**Figure 1:**
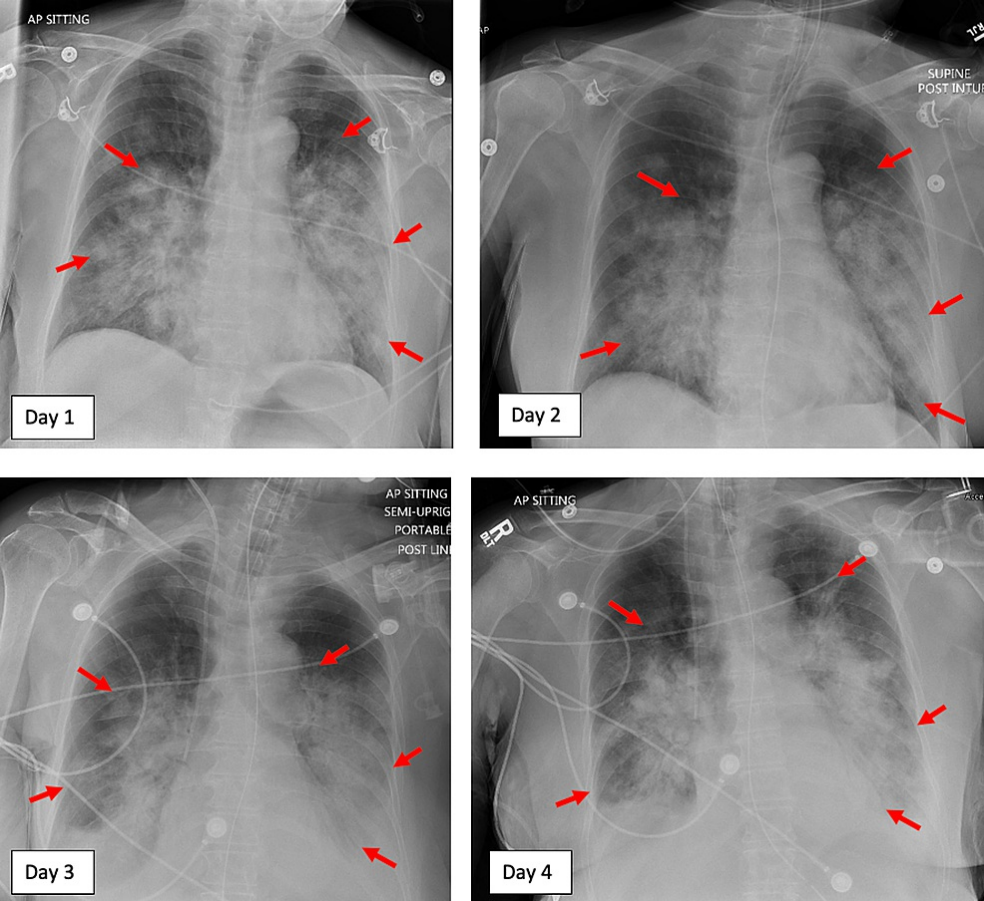
Sequential chest X-rays during the patient’s four-day hospitalization. The patient’s bilateral pulmonary infiltrates expanded over the four-day period. Her bilateral pleural effusions are marked with red arrows.

Prior to ingestion of gasoline, all laboratory values were within normal limits based on previous results in her healthcare record. However, abnormal laboratory findings at the time of admission included hypocapnia (21 mmol/L), lactic acidosis (2.4 mmol/L), elevated anion gap (13 mmol/L), elevated creatinine (1.46 mg/dL), decreased glomerular filtration rate (38 mL/min/1.73 m^2^), elevated aspartate aminotransferase (86 U/L), elevated total bilirubin (2.4 mg/dL), low protein (5.8 g/dL), leukocytosis (21.3 x 10^3^ cells/μL) and polycythemia (17.8 g/dL) (Tables 1-3). Initial chest X-ray revealed bilateral lung opacities suggestive of extensive bilateral consolidations secondary to aspiration pneumonitis (Figure 1).

**Table 1:** Serum K+, blood urea nitrogen, creatinine, anion gap, and glomerular filtration rate trends over the four-day hospitalization. The patient’s electrolyte status, acid-base status, and renal function deteriorated despite being supplied with intravenous fluids, ampicillin/sulbactam, ketamine, low-tidal volume ventilation, and hemodialysis. The upward arrows represent a value greater than the normal limit. The downward arrows represent a value lower than the normal limit.

	Day 1, 19:11	Day 1, 23:36	Day 2, 03:29	Day 2, 22:34	Day 3, 07:42	Day 4, 00:49
Serum K+	4.2	4.2	4.0	4.8	5.2 (↑)	5.7 (↑)
Blood Urea Nitrogen	13	15	19	32 (↑)	39 (↑)	26 (↑)
Creatinine	1.46 (↑)	1.73 (↑)	2.51 (↑)	4.11 (↑)	5.22 (↑)	4.04 (↑)
Anion Gap	13 (↑)	10	12	12	15 (↑)	22 (↑)
Glomerular Filtration Rate	38 (↓)	31 (↓)	20 (↓)	11 (↓)	8 (↓)	11 (↓)

**Table 2:** Arterial pH trend over four-day hospitalization. The patient’s acid-base status was unable to be restored to within normal limits. The downward arrows represent a value lower than the normal limit.

	Day 1, 20:38	Day 2, 06:19	Day 3, 04:23	Day 3, 06:52	Day 4, 00:12	Day 4, 06:18
Arterial pH	7.26 (↓)	7.34 (↓)	7.25 (↓)	7.22 (↓)	7.21 (↓)	7.23 (↓)

**Table 3:** Lactic acid trend over four-day hospitalization. Her lactic acid was unable to be restored to within normal limits despite supportive measures. By day 4, she developed critical lactic acidosis. The upward arrows represent a value greater than the normal limit.

	Day 1, 19:11	Day 1, 22:31	Day 2, 02:09	Day 2, 10:08	Day 2, 13:33	Day 2, 21:15	Day 3, 01:53	Day 3, 07:42	Day 3, 10:29	Day 4, 00:49	Day 4, 09:04
Lactic Acid	2.4 (↑)	2.3 (↑)	3.8 (↑)	2.1 (↑)	3.5 (↑)	3.5 (↑)	2.2 (↑)	2.3 (↑)	3.6 (↑)	12.0 (↑)	1.5

On the second day of admission, the patient developed worsening respiratory distress that required intubation and hyperventilation to accelerate elimination of volatile hydrocarbons. Supportive treatment consisted of intravenous fluids, ampicillin/sulbactam, ketamine, and adjusting the ventilator settings to low tidal volume ventilation, as set by the protocol developed by the National Institutes of Health Acute Respiratory Distress Syndrome Network [6].

Despite the appropriate supportive measures, the patient developed ongoing hypotension that failed to respond to intravenous fluids and pressors. This contributed to her worsening renal function and eventual oliguric acute renal failure. Her respiratory status further declined on the third day of admission such that she required a fraction of inspired oxygen of 95%. Severe acute respiratory distress syndrome could not be delayed, and extensive bilateral pleural effusions developed concomitantly (Figure 1). The patient’s condition was further complicated by minimal withdrawal to pain and sluggish pupillary reflexes consistent with acute toxic encephalopathy. Skin examination revealed diffuse petechiae across the abdomen and second-degree burns on the buttocks. Her electrocardiogram revealed a prolonged QTc interval (547 milliseconds).

**Figure 2:**
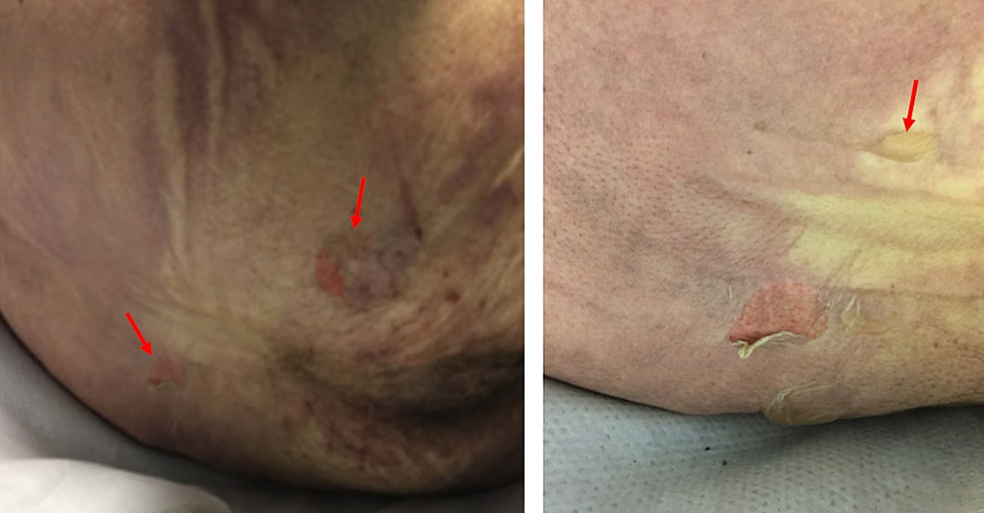
Cutaneous manifestations of gasoline ingestion. The gasoline passed through the patient’s entire gastrointestinal tract. Two small partial thickness wounds and one blister were present over the patient’s buttocks as indicated by the left and right images, respectively. The cutaneous lesions are marked with red arrows.

Her condition further worsened on day 4 when she developed critical lactic acidosis. The skin on her buttocks began to slough off. There were also two small partial thickness wounds and one intact blister (Figure 2). Given her worsening renal function and acidosis, hemodialysis was attempted but was unable to prevent multiorgan failure. It was decided to proceed with comfort care measures, and the patient passed away later that afternoon.

## Discussion

Hydrocarbons are ubiquitous environmental pollutants, and their exposures are common. Among the different types of hydrocarbons, there is a scarcity of case reports on gasoline poisoning in the current medical literature. Still yet, among reported cases of gasoline intoxication, the majority involve inhalation or percutaneous aspiration [7]. This highlights the need to describe cases and rationalize complications involving gasoline ingestion.

It has been established that the principal toxicity of gasoline ingestion is aspiration pneumonia [3,8,9]. Hydrocarbons in gasoline disrupt surfactant secretion by type II pneumocytes in turn reducing pulmonary compliance. Their presence is detected as foreign which triggers an acute inflammatory process, edema, and desquamation of the alveolar epithelium [9]. The resultant hypoxia occurs due to a combination of shunt formation, bronchospasm, and ventilation-perfusion mismatch. This presents symptomatically as cough, vomiting, nausea, tachypnea, and tachycardia. Breath sounds can either be normal, or there can be the presence of rhonchi, wheezing, or rales.

Any documented extra pulmonary manifestations of this condition may be important in the overall management of these patients. Hydrocarbons in jet fuel and other petroleum fuels are defatting agents which leads to dissolution of lipids in the skin and can result in mild inflammation or more serious chemical burns [10]. Prior studies suggested that hydrocarbons sensitize the myocardium to catecholamines which may be enough to cause arrhythmia such as prolonged QTc interval [11]. Toluene within gasoline often results in acute metabolic acidosis, hyperchloremia, hypokalemia, and acute tubular necrosis due to inhibition of distal tubular cell proton channels [12]. Among those patients with hydrocarbon aspiration secondary to ingestion, one-third have signs of central nervous system (CNS) toxicity ranging from drowsiness to stupor and seizures [13]. In this setting, the presence of CNS symptoms correlates strongly with the development of fever, hypoxemia, and pneumonitis [3,13].

Radiographic findings following gasoline poisoning vary. Ninety percent of patients with pulmonary complaints also had abnormal radiographs on arrival. Still yet, 50% of asymptomatic patients have abnormal chest radiographs, and of these patients with abnormal findings, 7% go on to develop significant toxicity [14]. Although this article discussed the findings in relation to childhood ingestions, similar generalizations can be made in adulthood ingestions. Additionally, chest radiographs can reveal areas of atelectasis consolidation, fibrosis, ground-glass opacities, or pleural effusions that can progress to acute respiratory distress syndrome [3,14].

After recognizing the complications of gasoline ingestion, rapid initiation of external and gastrointestinal decontamination should be prioritized regardless of age. External decontamination involves removal of contaminated clothing and cleaning affected hair and skin [15]. Gastrointestinal decontamination involves nasogastric aspiration within 60 minutes of the ingestion. If done outside of this window, there is an increased risk of pulmonary aspiration during this procedure [16]. Notably, there is no role for activated charcoal in gastrointestinal decontamination because it does not bind well to hydrocarbons, and epinephrine should not be used during resuscitation to prevent precipitation of cardiac arrythmias [11,17].

## Conclusions

Acute hydrocarbon exposure with gasoline can present as a wide array of pathology including pneumonitis, dermatitis, arrhythmia, encephalopathy, and acidosis. Clinical effects can potentially be predicted by the constituents of gasoline, route of exposure, and dose. Intentional ingestion exposures with aspiration are associated with the least favorable outcomes. Much of this lies in lack of an antidote for gasoline poisoning. Early and aggressive symptomatic care in the intensive care unit may provide a favorable outcome while minimizing pulmonary sequelae. Future studies might aim to investigate outcomes, supportive interventions, and complications that arise from the different methods of gasoline intoxication.
